# Open-access smart blood pump platform for controlling extracorporeal membrane oxygenation^☆^

**DOI:** 10.1016/j.ohx.2025.e00644

**Published:** 2025-03-25

**Authors:** Gabriella Glomp, Michael Cortelli, Briana Bernicker, Matthew Bacchetta, Rei Ukita

**Affiliations:** a Department of Biomedical Engineering, Vanderbilt University, PMB 351631, 2301 Vanderbilt Place, Nashville, TN 37235-1631, United States; b Department of Cardiac Surgery, Vanderbilt University Medical Center, 5025 Medical Center East, 1215 21st Ave. S., Nashville, TN 37232, United States

**Keywords:** Extracorporeal membrane oxygenation (ECMO), Centrifugal blood pump, Pulsatile flow, Pump console, Open access, Low-cost

## Abstract

Clinical blood pump consoles for extracorporeal membrane oxygenation (ECMO) are poorly accessible to researchers due to their high cost. Furthermore, clinical machines are built and designed at a high level of information security, which limits their integration with third-party machines and software. These barriers hinder researchers from customizing blood pump consoles for their investigational needs, limiting innovations and advancements in the areas of blood pump automation and pulsation. To address these needs, we present on a programmable Smart Blood Pump console. This console can be assembled for under $200 and uses open-source tools including Arduino. Using this console, centrifugal blood pump heads can be operated at clinically relevant levels of flow and pressure needed in extracorporeal life support applications (>250 mmHg pressure head, >4 L/min of blood flow). Additionally, the programmable nature allows for utility beyond the standard indications of clinical extracorporeal blood pumps, including pulsatility and servo control. For future directions, this console will be further developed to accommodate a wider range of clinical pump heads. We envision that this will be an affordable, open-access platform to suit the varying needs of engineers and researchers for fostering innovations in ECMO technology.

## Graphical abstract

**Figure:**
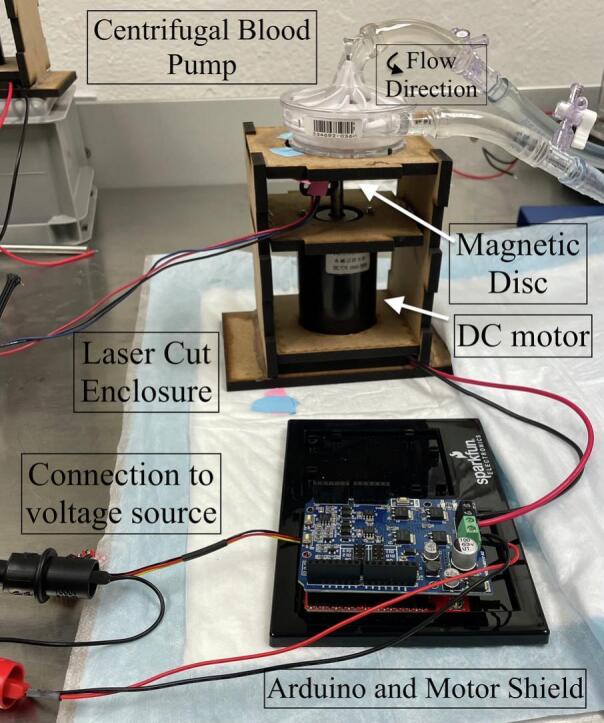

**Specifications Table:** 

Hardware name	Smart Blood Pump Platform
Subject area	Educational tools and open-source alternatives to existing infrastructure
Hardware type	Mechanical Engineering, Materials Science
Closest Commercial Analog	Bio-Console Pump Speed Controller
Open Source License	CC BY 4.0
Cost of Hardware	$166.13
Source File Repository	Zenodo: https://doi.org/10.5281/zenodo.12555206

## 1 Hardware in context

**Fig. 1:**
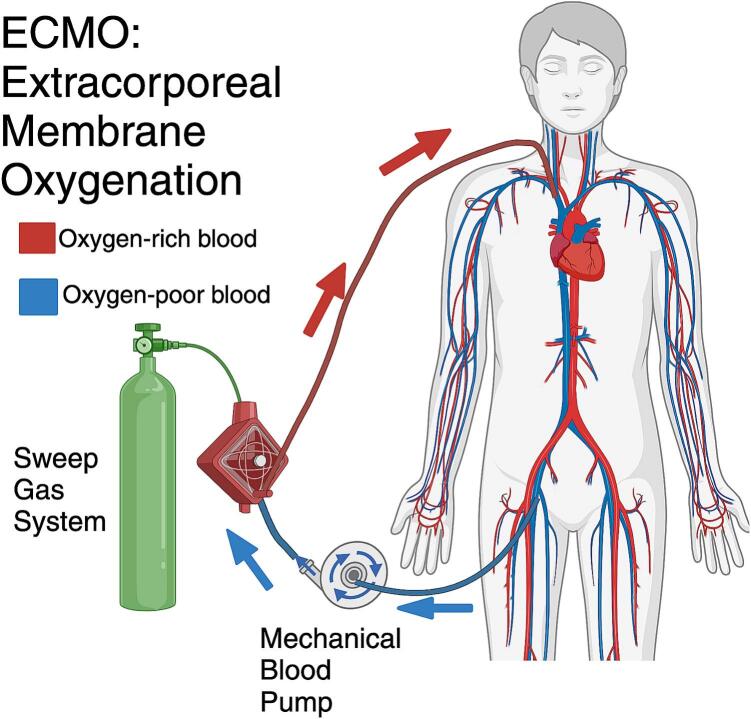
Diagram of veno-venous extracorporeal membrane oxygenation circuit. Created with BioRender.

The overarching goal of the project is to develop a low-cost, programmable console that can operate centrifugal blood pumps used in extracorporeal membrane oxygenation (ECMO). Patients suffering from cardiopulmonary failure, difficult post-operative recoveries after transplant, severe trauma, and infection may need ECMO support. ECMO consists of a mechanical blood pump and an oxygenator to provide extracorporeal physiologic support (Fig. 1). Patients can remain on ECMO from days to weeks as a bridge to recovery or to transplant. Between 2019 and 2024, there have been more than 102,000 ECMO runs, which makes up more than half of all reported cases since 1989 and demonstrates a rapid growth in the clinical demand for this technology [1]. Within this period, ECMO was especially important for supporting COVID-19 patients [2], [3]. However, the high resource cost of therapy necessitates rigorous patient selection for ECMO, and this led to potentially preventable deaths during the peak of the COVID-19 pandemic [3]. These limitations serve as a clinical motivation to innovate the enabling technology of ECMO to make the therapy less resource-intensive, particularly the labor cost. At an average hospital in the U.S., supporting a patient on ECMO costs $4,584 to $11,524 per day including materials, pharmaceuticals, machines, specialized personnel, procedures, and hospital stay [4]. Personnel cost can be up to 80 % of the total cost [5]. Bedside staff must be vigilant in assessing the patient's condition and ensuring that the ECMO system adequately supports the patient. This process, as it exists, is labor-intensive and time-consuming, strains healthcare worker availability, and increases susceptibility to errors. Semi-automating any part of the manual responsibilities will help decrease the labor cost and expand the access of care, which is especially important in the settings of respiratory disease outbreak and mass casualty events.

To advance research and innovation in this area, we need to address the barriers that may be hindering progress. Namely, standard clinical machines for operating ECMO − especially the blood pump console − are expensive. Clinical centrifugal pump consoles can cost more than $40,000 [6]. Furthermore, the high level of information security built into the clinical systems limits the user’s ability to program or integrate with a third-party device, which is crucial to implementing features such as automation. Even blood pump consoles solely for preclinical use can cost more than $10,000 [7]. A low-cost, programmable pump console would address this issue by making the technology more accessible.

Furthermore, a low-cost programmable pump console can also help advance research in the scientific area of pulsatility. Pulsatility in extracorporeal circulation has been a much-debated concept about its potential benefit over constant flow mode. There has been little research to prove the benefits of pulsatility in terms of improving end-organ perfusion, reducing clot burden inside the oxygenator, and improving gas transfer rates within the system and the body [8], [9]. Currently, the Novalung Heart & Lung system − specifically its DP3 diagonal pump − is the only clinically approved pump for extracorporeal applications that can provide pulsatile flow. Meanwhile, we have a limited understanding of how other clinical ECMO pumps function under a pulsatile setting.

Here, we designed and built a low-cost, do-it-yourself (DIY), Smart Blood Pump platform that can operate a clinical centrifugal pump at hemodynamic conditions relevant to extracorporeal applications. The programmable feature is also demonstrated by pulsatility and a simple servo control for maintaining flow. As the first pass, this work focused on one specific type of pump head − LivaNova Revolution. This prototype effort serves as the first iteration toward ultimately establishing a universal pump console that is affordable, operable across various types of pump heads, and programmable to fit the needs of engineers and clinicians.

## 2 Hardware description

**Fig. 2:**
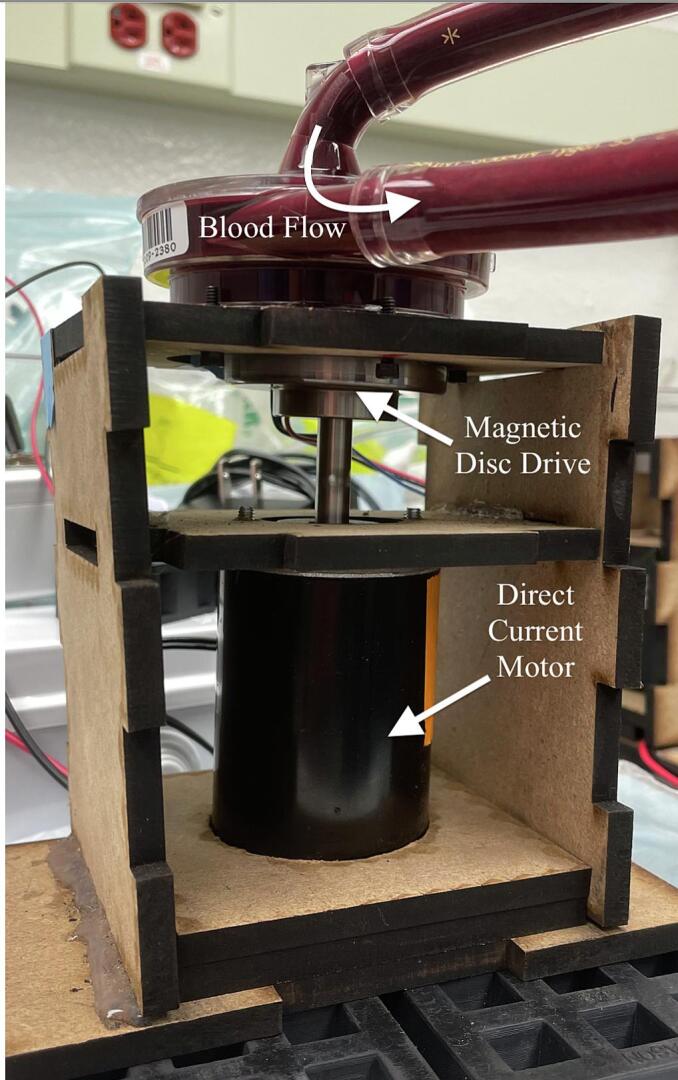
Assembled Smart Blood Pump platform with plywood panels*.*

Many of the clinical centrifugal blood pumps used for ECMO, including Medtronic Affinity and LivaNova Revolution, have impeller blades that are embedded with magnets to enable their contactless magnetic drive. The presented prototype for our console consists of a direct current permanent magnet motor, a magnetic disc that drives the pump impeller, and a custom-designed, laser-cut, wooden enclosure that houses the motor and the magnetic disc (Fig. 2). The enclosure is designed to ensure proper alignment and coupling between the magnetic disc and the pump impeller. The pump can be controlled manually by connecting the motor to a direct current (DC) variable power supply. For advanced, programmable flow control, the motor can be connected to an Arduino-based microcontroller and a motor driver for regulating the motor speed in a programmed way. For these intentions, we will provide Arduino scripts that demonstrate pulsatility and servo flow control (Table 1).

## 3 Design files

**Table 1:** Summary of design files.

Design file name	File type	Open source license	Location of the file
laser_cut_enclosure.dwg	.dwg	CC BY 4.0	Zenodo: https://doi.org/10.5281/zenodo.12555206
sinusoid_pulsatile_flow.ino	.inosoftware (Arduino IDE)	CC BY 4.0	Zenodo: https://doi.org/10.5281/zenodo.12555206
PID_flow_control.ino	.inosoftware (Arduino IDE)	CC BY 4.0	Zenodo: https://doi.org/10.5281/zenodo.12555206

## 4 Bill of materials

**Table 2:** Bill of materials summary for enclosure assembly.

Designator	Component	Quantity	Cost per unit	Total Cost- Currency	Source of Materials	Material Type
Enclosure	Plywood Sheets For Laser Cutter 12″ x 24″ x ⅛”	1	$4.60/count	$4.60	Provided by Vanderbilt Wondr’y Center. Materials also on Amazon	Organic Polymer
Enclosure	DC Motor, XD-3420	1	$18.69/each	$18.69	Amazon	NA
Enclosure	Magnetic Coupling Shaft (9199T2), 1–3/8″ Overall Length, 1–31/32″ OD (Magnetic Disc)	1	$101.67/each	$101.67	McMaster Carr	Metal
Electrical	SHIELD-MD10 Cytron 10A Motor Driver Shield	1	$14.50	$14.50	Maker Motor	NA
Electrical	SparkFun RedBoard − Programmed with Arduino	1	$21.50	$21.50	SparkFun	NA
Electrical	LittelFuse Hall Effect Sensor (54100-17X-02-A)	1	$5.17	$5.17	DigiKey	NA
Total			$166.13			

### 5 Build instructions

**Fig. 3:**
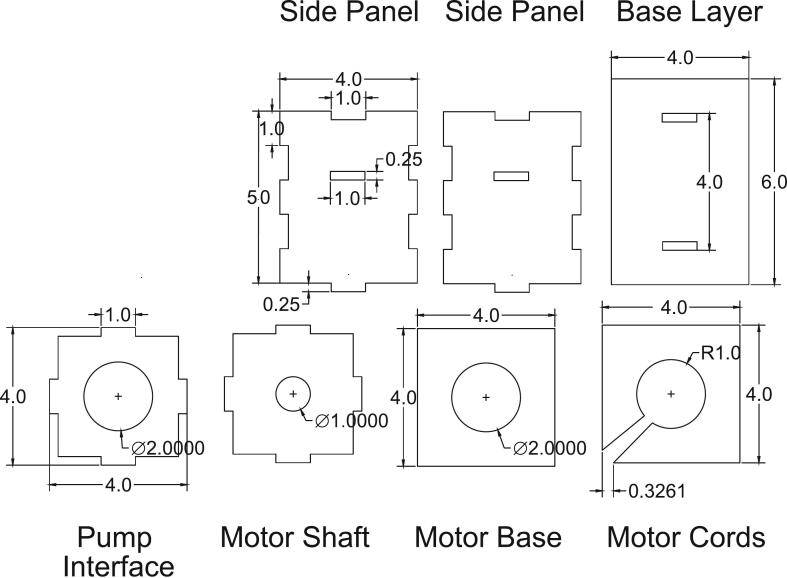
Two-dimensional computer-aided drawing of the enclosure panels for laser cutting. All annotated dimensions are in inches. For circular dimensions, R indicates the radius and Ø represents diameter. See the Zenodo repository for the actual file.

**Fig. 4:**
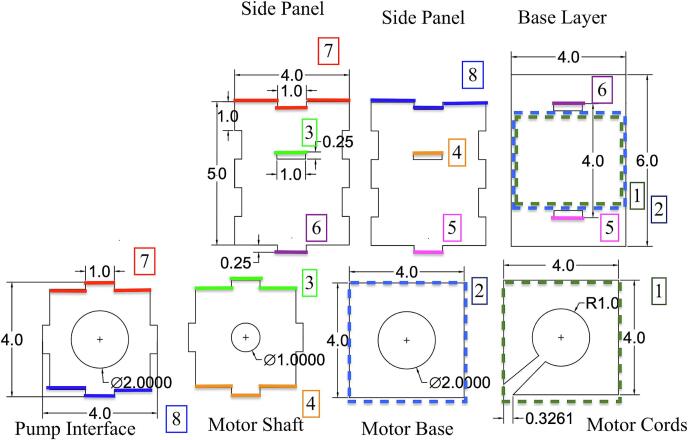
Assembly instructions for the enclosure. Color coding and numbers are provided to show how different panels should fit together. See the Zenodo repository for the actual file.

**Fig. 5:**
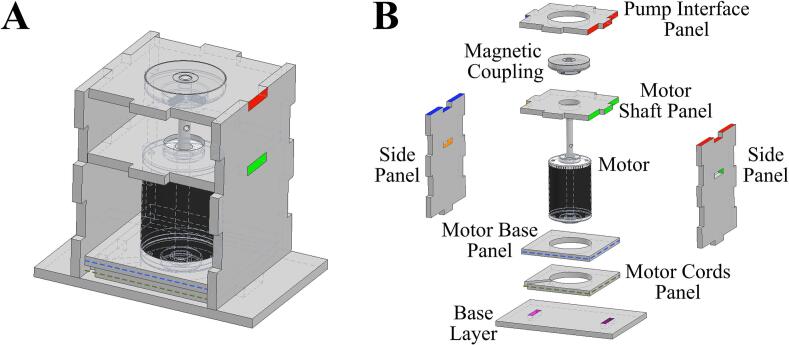
Three-dimensional rendering of A) the assembled Smart Blood Pump enclosure, pump, and magnetic coupling, and B) the exploded view of the assembly. Color coding is the same as Fig. 4.

**Fig. 6:**
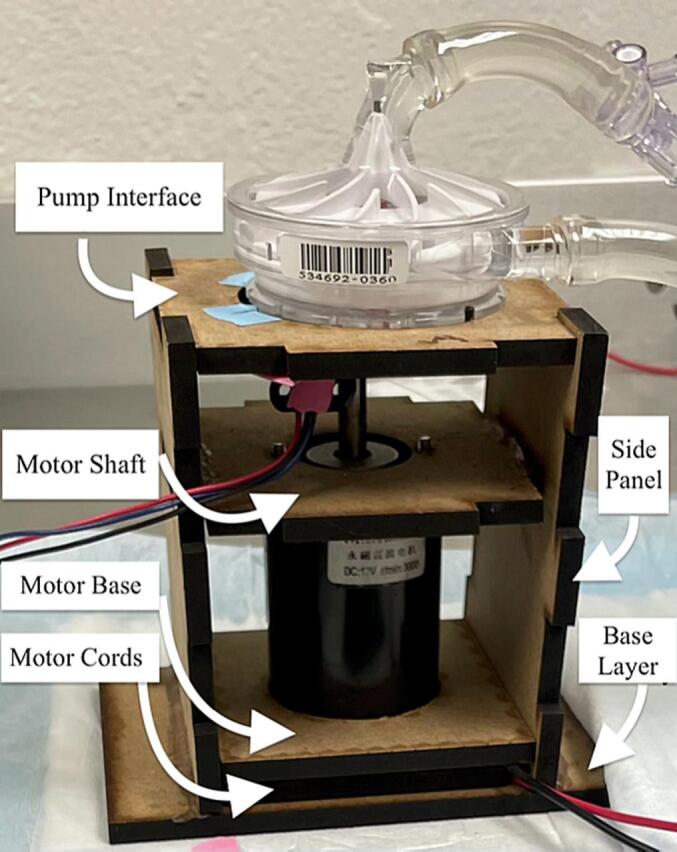
Assembled prototyped enclosure with wood sheets with the panels labeled.

Table 2 displays the Bill of Materials needed for the assembly of the enclosure. This enclosure consists of seven laser-cut panels – pump interface, motor shaft, motor base, motor cords, side panel (x2), and base panel. The panel dimensions were selected to provide the most optimal compatibility with the LivaNova Revolution pump. To allow the components to interlock, dents and holes were cut into the panels (Fig. 3, Fig. 4, Fig. 5, Fig. 6). The base panel is used for stabilizing the motor and have openings to insert the side panels. The distance between the magnet and the pump head should be maintained at 0.5 cm, so the total height of the side panels should account for the combined lengths of the motor, the shaft, and this ideal interface spacing. The pump interface panel’s cut-out should be large enough to accommodate the pump diameter.

For assembly, vertically insert the two side panels into the slots of the base layer panel (Fig. 4, Fig. 5, purple and magenta). Between the two side panels, slide the motor cords panel onto the top of the base layer panel (Fig. 4, Fig. 5, dotted green). Then place the motor base panel (Fig. 4, Fig. 5, dotted blue) on top of the motor cords panel. The DC motor is fitted through the center holes of the motor base and the motor cords panels. The diagonal slits in the motor cords panel can be used to tunnel out the motor cords. Next, slide the motor shaft panel between the two side panels and pass the shaft through the center hole of this panel. The magnetic disc can be directly coupled to the motor shaft using provided set screws. The pump interface panel is then fitted onto the top of the two side panels (Fig. 4, Fig. 5, red and blue). Finally, the pump head is placed on the pump interface panel and secured with three sets of bolts. Fig. 5, Fig. 6 show the completed assembly with the panels labeled. The enclosure's pump interface panel, where the pump head sits, is designed to be modular and generally compatible with magnetically driven centrifugal pump heads. Later we will discuss additional changes that need to be made to accommodate other pump types.

**Fig. 7:**
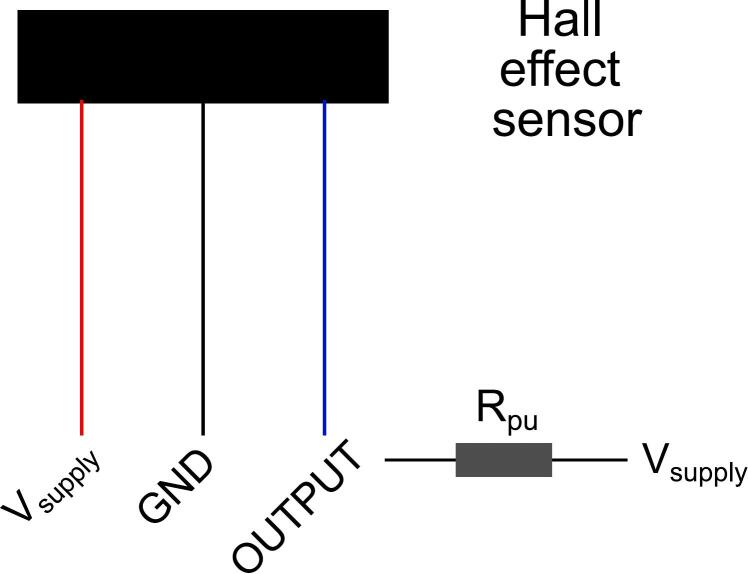
Hall effect sensor setup for measuring pump speed. R_pu_ = pull-up resistor.

*Electronic Setup*: A Hall effect sensor can be used to monitor the real-time rotational speed of the magnetic disc. This sensor should be fixed within the activating distance of 13 mm to the magnetic disc. Connect the red wire from the Hall effect sensor to a supply voltage of 5 V, and connect the black wire to ground (Fig. 7). Connect the blue wire to a data acquisition system (Fig. 7, see later for connecting to the PowerLab system). This blue data output wire should also be connected to a pull-up resistor of 200 O to the same 5-volt voltage supply.

**Fig. 8:**
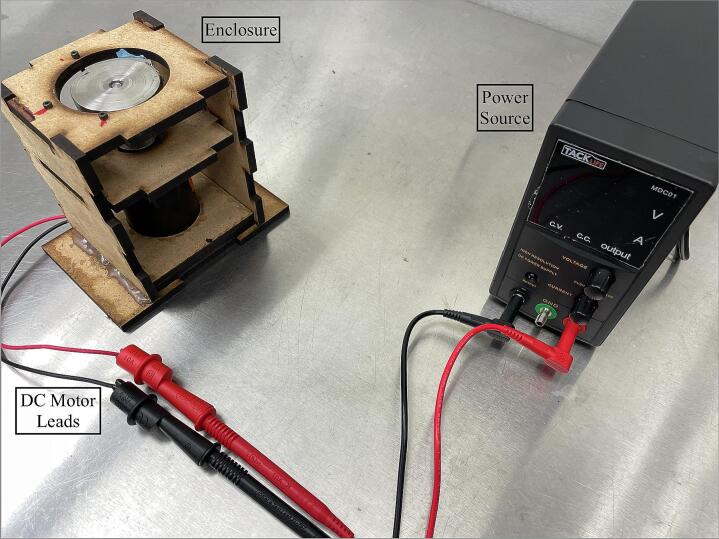
Electrical connection for manual control of the Smart Blood Pump using a variable voltage power supply.

The motor can be directly connected to a DC variable power supply to manually operate the pump. Connect the positive (red) and negative (black) terminals from the supply to the positive (red) and negative (black) leads of the motor, respectively, to rotate the magnetic disc in the direction of the LivaNova Revolution’s blood flow (Fig. 8).

**Fig. 9:**
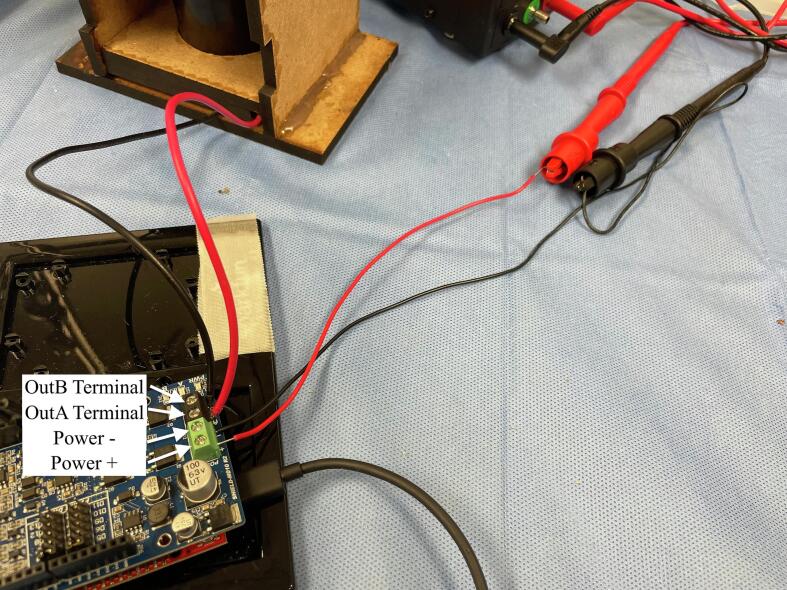
Screw terminals of the motor driver shield attached on top of the Sparkfun Redboard microcontroller are used for connecting to the input voltage source (green) and to the output voltage to the motor (black). (For interpretation of the references to color in this figure legend, the reader is referred to the web version of this article.)

**Fig. 10:**
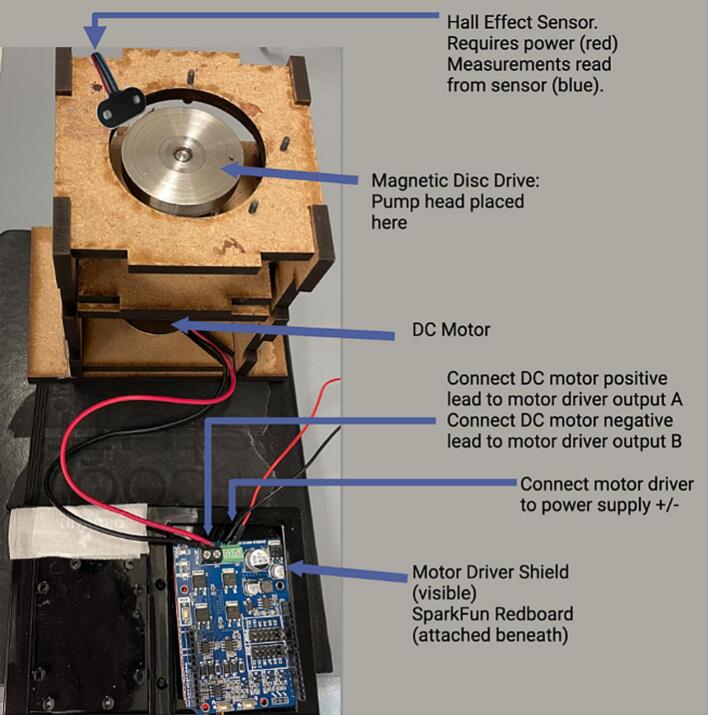
Bird’s eye view of the programmable control setup of the Smart Blood Pump using the Sparkfun Redboard microcontroller and its Cytron motor driver shield.

Fig. 9 shows how the pump can be connected to the Sparkfun Redboard microcontroller for programmable flow. A Cytron motor driver shield was attached on top of the Redboard. To match with the blood flow direction of the LivaNova Revolution, attach the DC motor’s positive red terminal lead to the “outA” screw terminal pin of the motor driver shield, and the DC motor’s negative black terminal lead to the “outB” screw terminal pin. Also on the motor driver shield, locate the green screw terminals of the shield labeled “POWER” and its positive and negative terminals. Connect the positive screw terminal from the shield to the positive terminal of the power supply, and the negative screw terminal from the shield to the negative terminal of the power supply. *CAUTION: switching the polarity here may lead to damage of the motor driver shield* Fig. 10 shows the bird’s eye view of the programmable flow setup. Then, proceed to compiling and uploading an Arduino script to the Redboard for programmable operation of the pump.

**Fig. 11:**
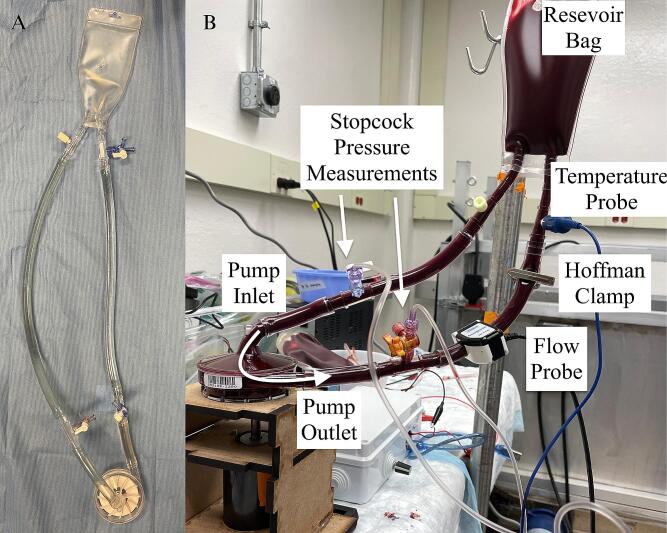
A) Constructed flow circuit loop using the LivaNova Revolution pump, B) and the circuit primed with bovine whole blood.

*Flow Circuit Loop*: The basic pump functionality can be confirmed using a simple flow loop circuit. The circuit design was published previously [10], [11] and consists of the following components in the specified order of assembly (Fig. 11A, Table 3): a two-port 100 mL reservoir bag, ¼” to ⅜” adapter with a luer sideport, 6″ of ⅜” tubing, ⅜- ⅜ connector with a luer sideport with a stopcock for pump inlet pressure, 6″ of ⅜” tubing, LivaNova pump head, 6″ of ⅜” tubing, ⅜-⅜ luer connector with a luer sideport and a stopcock for pump outlet pressure, 6″ of ⅜” tubing, ¼” to ⅜” adapter with a luer sideport and a temperature probe (Fig. 11B). Prime the circuit with at least 200 mL of fluid, which could be saline, 40 % glycerol solution to match the viscosity of blood, or whole blood if there is proper biohazard handling plan in place at your research site. Use the two three-way stopcocks within the flow circuit loop as access points for syringes to push the fluid in and to evacuate air out of the circuit. Once the circuit is primed and free of air bubbles, position the reservoir bag at an elevated height. An adjustable Hoffman clamp should be placed distally to the pump outlet pressure port for controlling the pump afterload. A Transonic flow probe can also be positioned, matching the direction of flow, to acquire a real-time flow signal.

**Table 3:** Summary of items for constructing the flow loop circuit. Asterisks (*) indicate that the exact volume will need to be adjusted according to the circuit volume as well as the target viscosity of the fluid.

Designator	Component	Quantity	Source of Materials	Material Type
Circuit Material	Glycerol* >99 %	1000 mL*	Fisher Chemical	glycerol
Circuit Material	Normal saline*	1000 mL*	ICU Medical	physiologic saline
Circuit Material	Two Port 100 mL Reservoir bag	1 per circuit	Qosina	polymer
Circuit Material	¼” to ⅜” luer adapter (0.0063 m to 0.019 m)	2 per circuit	Qosina	polymer
Circuit Material	⅜” to ⅜” luer adapter (0.019 m to 0.019 m)	2 per circuit	Qosina	polymer
Circuit Material	6″ (0.15 m) of Tygon tubing (inner diameter = 3/8″ (0.019 m), wall thickness = 3/32″ (0.0024 m)	4 segments	VWR, catalog #: 89404–282	polymer
Circuit Material	Three-way Stopcock	2 per circuit	Qosina	polymer
Circuit Material	LivaNova Revolution Pump Head	1 per circuit	eSutures	polymer
Circuit Material	Hoffman clamp	1 per circuit	McMaster-Carr	Metal

## 6 Operation instruction

*Manual Flow Mode*: After the circuit is primed and the motor is connected directly to a DC power supply as shown in Fig. 8, set the output voltage to a low level of 3 to 4 V. Ensure that the motor and the pump are rotating in alignment with the flow direction of the LivaNova Revolution pump. Then, adjust the power supply voltage according to the target flow, pressure, and/or rotational speed. Increase voltage in increments of 0.1 to 1 V to ensure a smooth change in speed. A large step change can yield a spike in the motor current that can lead to magnet decoupling.

*Programmable Flow Mode:* Navigate to Arduino IDE on your computer. IDE is available as an app or web-based interface (https://www.arduino.cc/en/software). Connect the RedBoard to a computer using the provided USB cable, and then select your board and port connection using the options from the IDE tools menu. We provide two different Arduino scripts to demonstrate programmed flow – pulsatile flow and servo control for flow adjustments (Table 1).

For pulsatile operation, compile and upload the Arduino script named “sinusoid_pulsatile_flow.ino” (Table 1). The script uses Arduino’s digital output pulse width modulation (PWM) to vary the input voltage to the motor. The PWM output value ranges from 0 to 255, where 0 represents zero voltage and 255 represents the full voltage equal to the power supply voltage. Pulsatile control can be achieved by changing the *minPWM* and *maxPWM* variables defined in the Arduino script. The difference between the two variables will set the pulse height. Additionally, the time duration of a full pulsatile cycle will need to be defined under the variable name *period* in the unit of milliseconds. For a default setting, we set *period* of 1000 ms, *minPWM* of 150, and *maxPWM* of 200. We caution the readers to not make the period too short, and to not make the pulse height too high when they are just starting out. Either of these changes will require rapid change in motor speed that can lead to magnetic decoupling. We recommend the readers to change these settings incrementally from the provided default values.

**Fig. 12:**
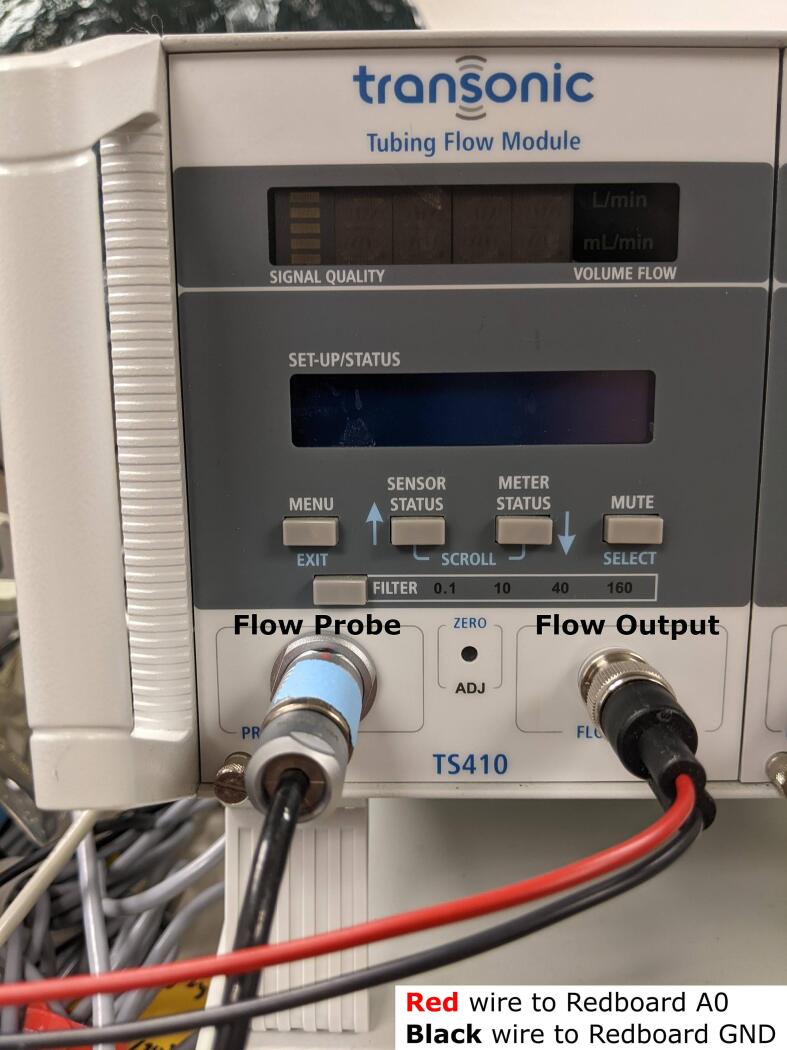
Transonic flow meter setup and wiring guideline to enable servo control of the flow rate with the Smart Blood Pump.

*Servo Flow Control*: To close the feedback loop between the Redboard and the flow sensor, you will also need to attach the Transonic flow probe onto one of the 3/8″ segments of the flow loop circuit. Ensure that the flow direction indicated on the probe matches with that of the circuit. Connect the other cable connection end of the flow probe to a pin labeled “PROBE” on the front panel of a Transonic flow meter (Fig. 12). Then, using a BNC-to-wire cable, connect the BNC end to the “FLOW OUTPUT” on the flow meter’s front panel. Connect the opposite wire ends of this cable to the Sparkfun Redboard − red wire to an analog input pin A0 of Redboard, and black wire to one of the ground (GND) pins on the Redboard (Fig. 12).

Compile and upload the Arduino script named “PID_flow_control.ino” (Table 1) to your Redboard. This script will demonstrate servo control of the pump flow using a proportional-integral-derivative control. To define a setpoint flow for the servo control, be sure to refer to your Transonic flow probe to correctly convert between flow and the corresponding voltage signals. For the ME9PXL flow probe used in this study, there is a scaling factor of 100 to convert from flow rate in L/min to the corresponding digital value between 0 and 1023 read by the Redboard (e.g. 3 L/min will be read as a digital value of 300). Use this scaling factor to define the value for the variable *Setpoint* in the Arduino script, which corresponds to the proportional integral derivative (PID) controller’s setpoint. The gain constants for proportional (variable is named *Kp* in the Arduino script), integral (*Ki*), and derivative (*Kd*) terms can also be adjusted to optimize for stability and oscillation. The provided script uses default values of 0.2, 0.5, and 0.1, respectively.

## 7 Validation and characterization

### 7.1 Methods

*Pressure-Flow Curves*: Centrifugal pumps are characterized by the level of pressure and flow they can generate to achieve the hydraulic requirement. This relationship can be visualized as pressure-flow (HQ) curves at varying speeds or voltage input levels. The hydraulic requirements of adult ECMO can vary depending on patient size and disease indication but typically operate at a pressure head between 250 and 300 mmHg and flow rate between 4 and 6 L/min [12], [13]. HQ curves were generated for LivaNova Revolution, Medtronic Affinity, and Spectrum Quantum pump heads using the described flow loop circuit (Table 3, Fig. 11) with a 40 % glycerin-saline mixture to match the viscosity of whole blood. The pump was operated between 6 and 12 V at a 1-volt increment. Pressure head, circuit flow, rotational speed, and motor current and voltage were acquired to quantitatively evaluate pump performance. Pressure head is defined as the pressure difference between the outlet and the inlet of the pump. An adjustable Hoffman clamp was used to control pump afterload to acquire an HQ curve for each input voltage level.

*Hemolysis*: Blood pumps can cause mechanical shear damage to cells over time, leading to hemolysis. To assess whether the console here causes any excessive unexpected levels of hemolysis, the pump console was evaluated over a 6-hour study at constant clinical pressure and flow conditions. The standard testing guideline by ASTM F1841-19: Standard Practice for Assessment of Hemolysis in Continuous Flow Blood Pumps was used [14]. Bovine blood was procured from a local slaughterhouse (Anderson Meat and Processing) and heparinized at a concentration of 4000–6000 units/liter.

After assembling the flow loop circuit as detailed in Fig. 11 and Table 3, the circuit was initially primed with 200 mL of physiologic saline and circulated for at least 5 min to wet all blood-contacting surfaces. The flow loop circuit was then drained of saline completely and primed with 200 mL of bovine blood. The pump afterload and motor voltage were adjusted to achieve of 4.2 L/min of flow and 250–300 mmHg of pump pressure head. The circuit was run at the target setting for 5 min to equilibrate, and then a 6-hour test was started at this time of t = 0 h.

Every two hours, 1 mL of blood was also drawn to measure glucose, hematocrit, pH, and hemoglobin using a point-of-care blood gas analyzer. Every hour, a 2-mL blood sample was drawn from the circuit to measure plasma-free hemoglobin following Cripps’ method [15], [16]. For this method, blood samples were first centrifuged at 1500g for 20 min. The plasma’s absorbance profile at light wavelengths of 576.5 nm ($A_{576.5})$, 560 nm ($A_{560})$, and 593 nm ($A_{593})$ were measured using SynergyHTX by BioTek, and these values were used to calculate plasma-free hemoglobin, in mg/dL, using Eq. (1) [16]::

(1) $$177.6\times \left[A_{576.5}-\frac{\left(A_{560}+A_{593}\right)}{2}\right]$$

Then, the normalized index of hemolysis (NIH) was calculated using Eq. (2) [17]:

(2) $$NIH\left(\frac{g}{100L}\right)=\Delta freeHb\times V\times \frac{100-Ht}{100}\times \frac{100}{Q\times \Delta T}$$

where Δ*freeHb* is the change in plasma free hemoglobin concentration (converted to g/L blood) relative to t = 0 h time point, V is the circuit blood volume in L, Q is the blood flow rate in L/min, Ht is the blood hematocrit (%), and ΔT is the time elapsed in minutes.

To verify that there is no background hemolysis under a static condition, a parallel static control testing was run by priming a bag with the same volume of blood as the pump testing and assessing for hemolysis and blood gas profile at the same specified time points.

**Fig. 13:**
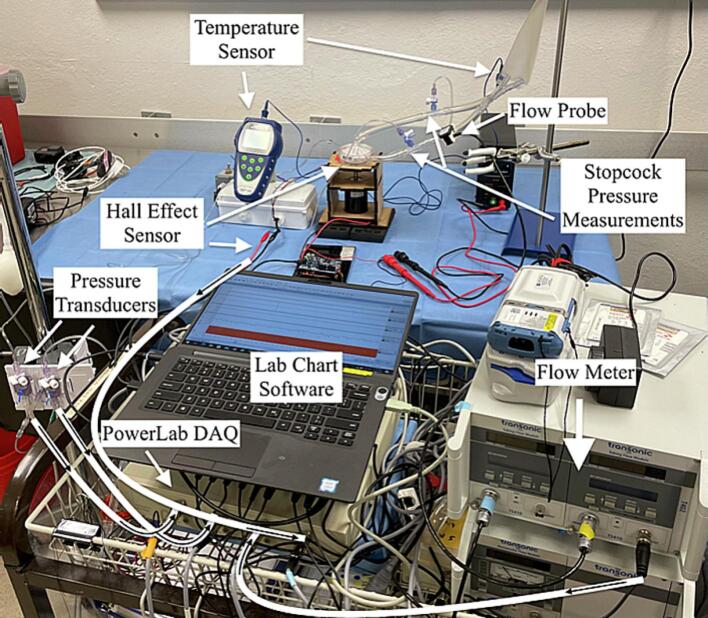
Data acquisition setup for monitoring flow, pressure, temperature, and pump speed during flow circuit loop testing.

*Data Monitoring*: Table 4 summarizes the data acquisition setup used to generate the validation data. Fig. 13 shows the data acquisition setup during hemolysis testing. Detailed instructions on assembling a similar data acquisition setup and calibrating the sensors can also be found in a prior publication [18]. Pump pressure head, Hall effect sensor and the corresponding motor/pump speed, and flow signals were all processed and recorded using the PowerLab and LabChart data acquisition system at a sampling frequency of 20,000 Hz. Individual fluid-filled pressure transducers were used to measure pump inlet and outlet pressures separately. Bridge amp front-end interface for PowerLab was used to receive the pressure transducer signals using Fogg System’s interface cables. Male-to-male BNC cables were then used to connect from the bridge amp front end’s output to the PowerLab’s analog input. For pressure transducers, a two-point calibration at 0 and 80 mmHg was manually performed using a sphygmomanometer. Flow was measured using a tubing clamp-on probe and a flow meter discussed earlier. A male-male BNC cable was used to connect from the Transonic flow meter’s flow output to an analog input channel of PowerLab. The flow probe was then calibrated in the LabChart software following the manufacturer's instructions for a two-point calibration between zero and full volt. Temperature was measured using a luer connector sensor and a temperature monitor every hour during hemolysis testing. The motor voltage and current were recorded from the DC variable power supply every hour during hemolysis testing. Pump speed was measured using a Hall effect sensor detailed earlier (Fig. 7). The Hall effect sensor’s output and ground wires were connected to the alligator clip end of the alligator-to-male BNC connector cable and fed to one of the PowerLab analog input channels using the BNC end. The raw output signal from the Hall effect sensor is a square pulse wave. For the presented magnetic coupling disc, four square pulses from the Hall effect sensor correspond to one full rotation of the disc. This raw square wave signal can be converted to rotations per minute by dividing the measured frequency of this square wave by 4 and multiplying by 60.

**Table 4:** Summary of data acquisition equipment and sensors for pressure-flow curve and hemolysis tests.

Designator	Component	Quantity	Source of Materials	Material Type
Electronic	200 Ohm Resistor, Jumper Wires Kit	1 Kit	Amazon	NA
Enclosure	Hall Effect Sensor 55100-3M-02-A	1	DigiKey	NA
Data Acquisition	Flow Tubing Clamp-On Probe ME9PXL	1	Transonic	NA
Data Acquisition	Flow Meter TS410	1	Transonic	NA
Data Acquisition	Fluid Filled Pressure Transducer TruWave	2	Edwards Lifesciences	NA
Data Acquisition	Temperature Luer Connector Sensor TM-TEMP-340	1	PendoTech	NA
Data Acquisition	Data Acquisition and Analysis System PowerLab	1	ADInstruments	NA
Data Acquisition	Bridge Amplifier Quad FE224	1	ADInstruments	
Data Acquisition	Interface cable between TruWave Edwards Lifesciences and Bridge Amp Catalog # 0395-2521	2	Fogg System	
Data Acquisition	Arterial Blood Gas Machine ePOC	1	Heska	NA
Data Acquisition	Male-Male BNC coaxial cable 3272-CO-058BNCX200-004-ND	1	Digi-Key	NA
Data Acquisition	Alligator clip-to-Male BNC connector cable 1286-1232-ND	1	Digi-Key	NA
Data Acquisition	BNC-to-wire cable 501-1031-ND	1	Digi-Key	NA

### 7.2 Results

**Fig. 14:**
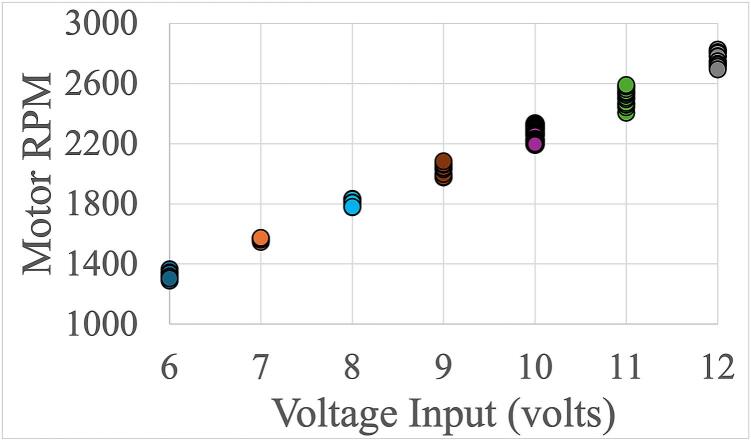
Pump speed in rotations per minute (RPM) achieved for voltage input using the LivaNova Revolution. Each data point represents the motor speed measured at different levels of circuit occlusion and corresponds to individual data points shown in Fig. 15’s pressure-flow profile.

**Fig. 15:**
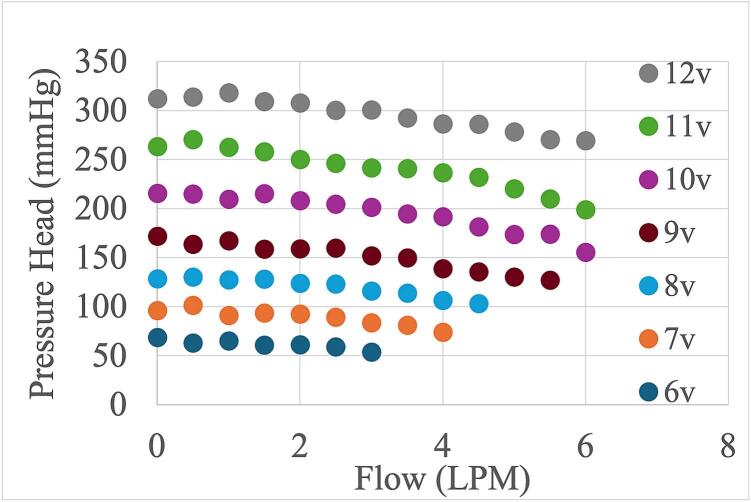
Pressure-flow curves of the LivaNova pump driven by the Smart Blood Pump platform using 40% glycerol solution circuit and input motor voltages between 6 and 12 V.

*HQ Curves:* The LivaNova Revolution pump achieved pump speeds between 1300 and 3000 RPM for voltage inputs between 6 and 12 V (Fig. 14). Slight variations in the RPM for a fixed input voltage were due to variations in the pump afterload, where higher pressure head/lower flow correlated with faster pump speed, and lower pressure/faster flow correlated with lower pump speed. At 12 V, the pump met the clinically relevant levels of flow and pressure for extracorporeal applications at >250 mmHg and >4 L/min (Fig. 15). Two other clinical centrifugal pumps – Medtronic Affinity and Spectrum Medical Quantum CP − were tested but did not attain as high of pressure or RPM as the LivaNova Revolution (see the Zenodo repository for their HQ curves).

**Fig. 16:**
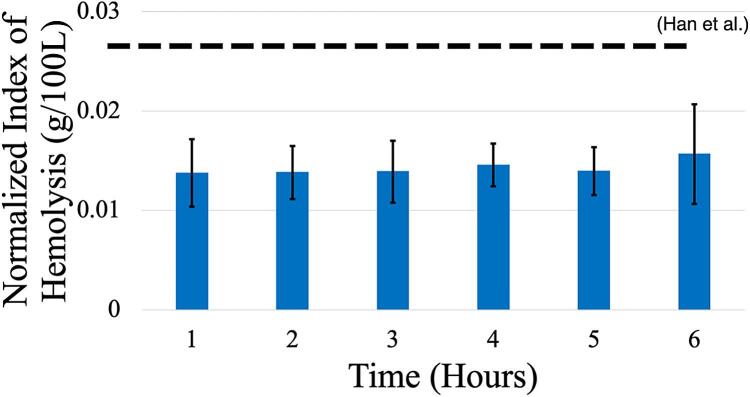
Normalized Index of Hemolysis (NIH) from blood flow loop test utilizing Smart Pump platform with LivaNova Pump head. Data shown as mean ± standard deviation (n = 3). Horizontal line in plot below represents average from the Han et al’s study.[19].

*Hemolysis*: The Smart Blood Pump console was assessed for pump biocompatibility, again using the LivaNova pump head as it achieved the most clinically relevant levels of flow and pressure. The acquired blood showed normal values prior to testing: the average hematocrit was 35 *±* 2 %, glucose was 117 *±* 9 mg/dL, and pH was 7.31 *±* 0.04 (n = 3). Average flow, pressure, hematocrit, temperature, and current during the 6-hour test are summarized in Table 5. The static control did not show any appreciable change in plasma-free hemoglobin over time. At a 6-hour mark, which serves as a benchmark for blood pumps, the normalized index of hemolysis was 0.014 *±* 0.0028 g hemoglobin/100 L blood (Fig. 16, n = 3). This value was a lower hemolytic index than a previously reported value that was calculated for a comparable but slightly higher flow and pressure profile (0.0287 *±* 0.0041 g/100 L) [19]. Therefore, our console shows comparable biocompatibility to that of a clinically approved console.

**Table 5:** Data summary from the six-hour hemolysis testing presented as mean ± standard deviation (N = 3).

	Hour 0	Hour 2	Hour 4	Hour 6
Hematocrit (%)	35 ± 2	33 ± 1	32 ± 1	31 ± 2
Glucose (mg/dL)	114 ± 5	81.3 ± 16.3	62 ± 17.5	45 ± 16.7
Temperature (C)	25.18 ± 0.97	37.67 ± 1.10	37.68 ± 0.96	37.53 ± 1.05
Motor Current (A)	1.7	1.7	1.7	1.7
Input Voltage (V)	12	12	12	12
Flow (L/min)	4.16 ± 0.1	4.16 ± 0.1	4.11 ± 0.05	4.09 ± 0.1
RPM	2776 ± 43	2782 ± 41	2773 ± 39	2776 ± 16
Pressure Head (mmHg)	274 ± 9	280 ± 14	281 ± 12	281 ± 14

**Fig. 17:**
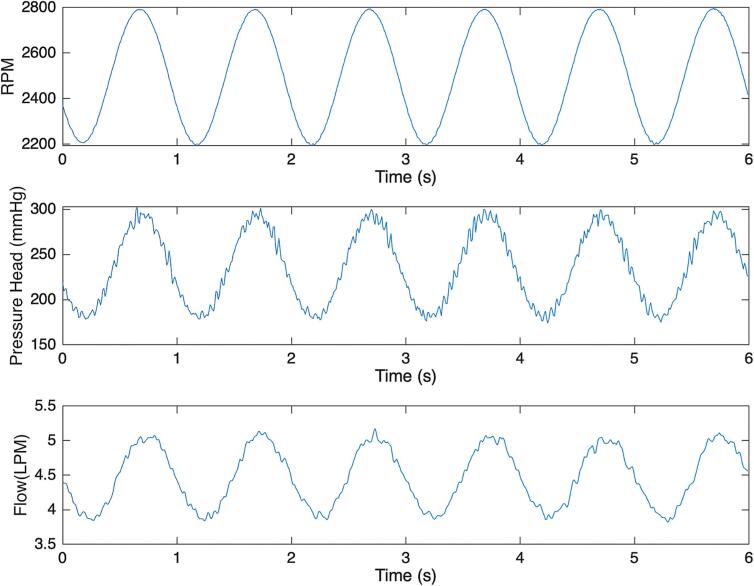
Representative pulsatile waveforms generated with the Smart Blood Pump using the following settings: input voltage of 13.5 V, Arduino variables *minPWM, maxPWM,* and *period* values of 180, 255, 1000 ms, respectively.

*Pulsatility of LivaNova Revolution:* Pulsatile speed, flow, and pressure were generated using LivaNova circuit and 40 % glycerol solution. Representative waveforms in Fig. 17 demonstrate oscillation between 2200 and 2800 RPM, 170 and 300 mmHg, and 4 and 5 L/min. These pulsatile profiles were achieved by adjusting the variables in the Arduino script titled “pulsatile_flow_control.ino”: *minPWM* and *maxPWM* of 180 and 255, respectively, and *period* of 1000 ms.

**Fig. 18:**
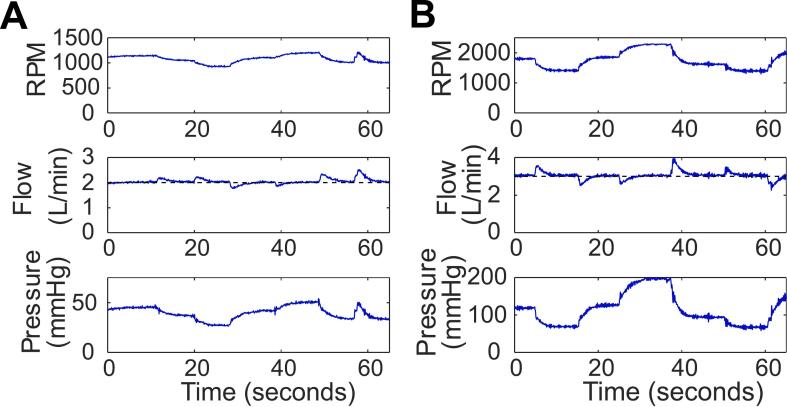
Servo control of the flow rate is implemented in the Smart Blood Pump to maintain setpoint flow rate of A) 2 L/min and B) 3 L/min.

*Servo Flow Control*: Fig. 18 demonstrates that the Smart Blood Pump can be servo controlled using PID algorithm to maintain target flow. The data were generated using *Kp*, *Ki*, and *Kd* values of 0.2, 0.5, and 0.1, respectively, in the Arduino script titled “PID_flow_control.ino.” The setpoint flow rate is shown as a dotted line for setpoint of 2 L/min in Fig. 18A and 3 L/min in Fig. 18B. The flow disturbances, caused by acute occlusion or release of the Hoffman clamp, are quickly corrected.

### 7.3 Conclusions and future directions

Future console design will need to expand its compatibility with other clinical pump head types to serve as a more universal platform, which is a lacking feature in many pump consoles in general but demonstrated by Anivia Extracorporeal Support System [20], [21]. The presented console and enclosure were most compatible with the LivaNova Revolution, and much less so with Medtronic Affinity or Spectrum Medical Quantum. The Medtronic Affinity pump has a thicker casing at the bottom which likely reduced torque transmission from the magnetic disc to its impellers. The Spectrum Medical Quantum pump’s impeller diameter is smaller than either Medtronic Affinity or LivaNova Revolution. Due to its specific customization for the LivaNova Revolution, the fit was suboptimal for these other two pump head types. Further optimization and customization of our enclosure will likely improve the performance with these other pumps. For example, a more powerful motor or magnetic coupling disc can be used to overcome the larger separation distance. Future directions will also include testing or modification to the current console for driving single pivot bearing pumps, since LivaNova Revolution is an older-generation pump with a dual pivot design. Likewise, long-term durability of the driven pump needs to be studied closely. In particular, the pivot bearing may wear out more quickly than the manufacturer’s estimate since there are different coupling forces at play with our presented design, so future studies will assess these pumps for longer than 6 h. Another future direction is to use the Smart Blood Pump console to implement more advanced forms of feedback control using physiologic sensors [22], [23] compared to simply keeping a constant flow as demonstrated here. Designing a safe automation strategy for extracorporeal life support will require a programmable blood pump that can be tuned to optimize for stability and control, so this investigation is a logical extension of the presented work here.

In conclusion, our work demonstrated a rapidly available, inexpensive console to operate centrifugal blood pumps for extracorporeal life support applications. This technology will foster research and innovation in this scientific and clinical area to ultimately develop a smarter blood pump.

*Human and Animal Rights:* This work did not use human or animal subjects.

### CRediT authorship contribution statement

**Gabriella Glomp:** Writing – review & editing, Writing – original draft, Visualization, Validation, Investigation, Data curation. **Michael Cortelli:** Writing – review & editing, Resources, Investigation. **Briana Bernicker:** Writing – review & editing, Investigation. **Matthew Bacchetta:** Writing – review & editing, Validation, Resources. **Rei Ukita:** Writing – review & editing, Supervision, Resources, Project administration, Conceptualization.

## Declaration of competing interest

The authors declare the following financial interests/personal relationships which may be considered as potential competing interests: Dr. Rei Ukita currently has an active research contract with CDX Medical Technologies LLC (VUMC112907); however, this contract is unrelated to the work presented. Dr. Matthew Bacchetta is an equity owner of Advanced Respiratory Technologies, Inc. and has received material support from CDX Medical Technologies LLC.

## References

[1] ELSO International Summary of Statistics | ECMO | ECLS, (n.d.). https://www.elso.org/registry/internationalsummaryandreports/internationalsummary.aspx (accessed February 26, 2024).

[2] Schmidt M., Hajage D., Lebreton G., Monsel A., Voiriot G., Levy D., Baron E., Beurton A., Chommeloux J., Meng P., Nemlaghi S., Bay P., Leprince P., Demoule A., Guidet B., Constantin J.M., Fartoukh M., Dres M., Combes A., Luyt C.-E., Hekimian G., Brechot N., de Chambrun M.P., Desnos C., Arzoine J., Guerin E., Schoell T., Demondion P., Juvin C., Nardonne N., Marin S., D’Alessandro C., Nguyen B.-L., Quemeneur C., James A., Assefi M., Lepere V., Savary G., Gibelin A., Turpin M., Elabbadi A., Berti E., Vezinet C., Bonvallot H., Delmotte P.-R., Sarcus M.D., Tour C.D.F.D.L., Abbas S., Maury E., Baudel J.-L., Lavillegrand J.-R., Oufella H.A., Abdelkrim A., Urbina T., Virolle S., Deleris R., Bonny V., Marec J.L., Mayaux J., Morawiec E. (2020). Extracorporeal membrane oxygenation for severe acute respiratory distress syndrome associated with COVID-19: a retrospective cohort study. Lancet Respir. Med..

[3] Gannon W.D., Stokes J.W., Francois S.A., Patel Y.J., Pugh M.E., Benson C., Rice T.W., Bacchetta M., Semler M.W., Casey J.D. (2022). Association between availability of extracorporeal membrane oxygenation and mortality in patients with COVID-19 eligible for extracorporeal membrane oxygenation: a natural experiment. Am. J. Respir. Crit. Care Med..

[4] Oude Lansink-Hartgring A., van Minnen O., Vermeulen K.M., van den Bergh W.M. (2021). Dutch extracorporeal life support study group, hospital costs of extracorporeal membrane oxygenation in adults: a systematic review. PharmacoEconomics - Open 5.

[5] Mishra V., Svennevig J.L., Bugge J.F., Andresen S., Mathisen A., Karlsen H., Khushi I., Hagen T.P. (2010). Cost of extracorporeal membrane oxygenation: evidence from the Rikshospitalet University Hospital, Oslo, Norway. Eur. J. Cardiothorac. Surg..

[6] Wang S., Moroi M.K., Kunselman A.R., Myers J.L., Ündar A. (2020). Evaluation of centrifugal blood pumps in term of hemodynamic performance using simulated neonatal and pediatric ECMO circuits. Artif. Organs.

[7] Harvard Apparatus, Centrifugal Pump, (n.d.). https://www.harvardapparatus.com/catalog/product/view/id/8293/s/centrifugal-pump/category/522/ (accessed July 5, 2024).

[8] Schraven L., Kaesler A., Flege C., Kopp R., Schmitz-Rode T., Steinseifer U., Arens J. (2018). Effects of pulsatile blood flow on oxygenator performance. Artif. Organs.

[9] Sakurai H., Fujiwara T., Ohuchi K., Hijikata W., Inoue Y., Maruyama O., Tahara T., Yokota S., Tanaka Y., Takewa Y., Mizuno T., Arai H. (2023). Innovative experimental animal models for real-time comparison of antithrombogenicity between two oxygenators using dual extracorporeal circulation circuits and indocyanine green fluorescence imaging. Artif. Organs.

[10] Woelke E., Mager I., Schmitz-Rode T., Steinseifer U., Clauser J.C. (2021). Validation of a miniaturized test loop for the assessment of human blood damage by continuous-flow left-ventricular assist devices. Ann. Biomed. Eng..

[11] Woelke E., Klein M., Mager I., Schmitz-Rode T., Steinseifer U., Arens J., Clauser J.C. (2020). Miniaturized test loop for the assessment of blood damage by continuous-flow left-ventricular assist devices. Ann. Biomed. Eng..

[12] Tonna J.E., Abrams D., Brodie D., Greenwood J.C., Mateo-Sidron J.A.R., Usman A., Fan E. (2021). Management of adult patients supported with venovenous extracorporeal membrane oxygenation (VV ECMO): guideline from the extracorporeal life support organization (ELSO). ASAIO J..

[13] Gajkowski E.F., Herrera G., Hatton L., Velia Antonini M., Vercaemst L., Cooley E. (2022). ELSO guidelines for adult and pediatric extracorporeal membrane oxygenation circuits. ASAIO J..

[14] F04 Committee, Practice for Assessment of Hemolysis in Continuous Flow Blood Pumps, ASTM International, n.d. https://doi.org/10.1520/F1841-19.

[15] Cripps C.M. (1968). Rapid method for the estimation of plasma haemoglobin levels. J. Clin. Pathol..

[16] Malinauskas R.A. (1997). Plasma hemoglobin measurement techniques for the in vitro evaluation of blood damage caused by medical devices. Artif. Organs.

[17] Naito K., Mizuguchi K., Nosé Y. (1994). The need for standardizing the index of hemolysis. Artif. Organs.

[18] Ukita R., Stokes J.W., Wu W.K., Talackine J., Cardwell N., Patel Y., Benson C., Demarest C.T., Rosenzweig E.B., Cook K., Tsai E.J., Bacchetta M. (2021). A large animal model for pulmonary hypertension and right ventricular failure: left pulmonary artery ligation and progressive main pulmonary artery banding in sheep. J. Vis. Exp..

[19] Han D., Leibowitz J.L., Han L., Wang S., He G., Griffith B.P., Wu Z.J. (2022). Computational fluid dynamics analysis and experimental hemolytic performance of three clinical centrifugal blood pumps: revolution, Rotaflow and CentriMag. Med. Nov. Technol. Devices.

[20] Vercaemst L. (2024). Year in review: highlights in ECLS innovation and technology, Anno 2022-2023. Perfusion.

[21] ANIVIA SG1000 Pump Console, (n.d.). https://www.apmtd.com/products-solutions/ (accessed July 5, 2024).

[22] Conway R.G., Berk Z.B., Zhang J., Li T., Tran D., Wu Z.J., Griffith B.P. (2020). Evaluation of an autoregulatory ECMO system for total respiratory support in an acute ovine model. Artif. Organs.

[23] Spencer B.L., Shaikh N., Gudex L., Dann T., Langley M., Matich H., Bartlett R.H., Rojas-Peña A., Potkay J.A. (2023). In vivo testing of an ambient air based, portable, and automated CO2 removal controller for artificial lungs. ASAIO J..

